# Prolonged early-life antibiotic exposure alters gut microbiota but does not exacerbate lung injury in a rat pup model

**DOI:** 10.1038/s41390-025-03924-2

**Published:** 2025-04-09

**Authors:** Mi-Yun Hsueh, Mei-Jy Jeng, Chia-Sui Chou, Chia-Wei Chang, Ciao-Ting Zou

**Affiliations:** 1 Institute of Emergency and Critical Care Medicine, College of Medicine, National Yang Ming Chiao Tung University, Taipei, Taiwan, ROC; 2 Neonatal Medical Care Center, Department of Pediatrics, Taipei Veterans General Hospital, Taipei, Taiwan, ROC; 3 Department of Pediatrics, School of Medicine, National Yang Ming Chiao Tung University, Taipei, Taiwan, ROC

## Abstract

**Background:**

Early antibiotic exposure may disrupt gut microbiome and affect the gut-lung axis. We examined the impact of prolonged antibiotic exposure during early life on growth and subsequent acute lung injury (ALI) in a rat pup model.

**Methods:**

Thirty-four 7-day-old rat pups were divided into Control, Antibiotics (Anti), Lung injury (LI), and Antibiotics-Lung Injury (Anti-LI) groups. The Anti and Anti-LI groups received oral Amoxicillin-Clavulanic acid from 7 to 40 days old, while Control and LI groups received sham water. ALI was induced in LI and Anti-LI groups with intratracheally administered lipopolysaccharide at 41 days old; all were sacrificed at 42 days old. Fecal bacterial sequencing, serum cytokine analysis, and pulmonary histological examination were performed.

**Results:**

Control and LI groups showed better weight gain from day 19 compared to Anti and Anti-LI groups. Anti and Anti-ALI groups exhibited decreased fecal microbial diversity (*P* < 0.05) and reduced *Firmicutes* abundance (*P* < 0.05) versus Control and LI groups. No significant difference in ALI severity was found between antibiotic-treated and non-treated groups.

**Conclusions:**

Prolonged early-life antibiotic exposure in this rat pup model significantly reduced gut microbiota diversity and exhibited a non-significant trend toward lower weight gain, without exacerbating the severity of subsequent LPS-induced ALI.

**Impact:**

- Prolonged early-life antibiotic exposure decreased gut microbial diversity in rat pups. Antibiotics-exposed groups exhibited a trend of reduced weight gain compared to controls, although the difference was not statistically significant.
- Despite the observed alterations in the gut microbiota, there were no significant differences in the severity of subsequent acute lung injury between the groups with and without prolonged antibiotic exposure.
- The study findings advocate for a more judicious use of antibiotics in neonates, emphasizing that appropriate antibiotic stewardship is critical for preserving gut health and may also support growth.

## Introduction

Critically ill neonates are highly vulnerable to infections due to immature immune systems and a lack of maternal antibodies.^1^ While antibiotics are commonly used to treat and prevent infections, prolonged use can cause diarrhea, drug resistance, and gut microbiota disruption.^2,3^ Prenatal and postpartum antibiotic use in preterm infants further alters their gut microbiota.^4,5^ High-risk infants are susceptible to extended antibiotic use. Therefore, the impacts of prolonged early-life antibiotic exposure warrant careful examination.

Researches on the gut-lung axis has highlighted immune cell interactions involved in dysbiosis.^6^ Dysbiosis may disrupt systemic immunity and lung inflammation.^7,8^ Understanding gut microbiota’s influence on the lung microbiome is vital for clarifying pulmonary disease progression in infants with immature immune systems.

Animal studies indicate that manipulating gut microbiota can worsen allergic airway inflammation.^9^ Early-life antibiotic exposure alters gut microbiota but has little effect on hyperoxia-induced lung injury.^10^ Data on previous antibiotic exposure’s impact on inflammation-induced ALI is sparse. In LPS-induced ALI, antibiotics and LPS instillation acutely change cecal and serum microbiota.^11^ Fecal microbiota transplantation (FMT) restores gut microbiota, reducing tissue damage in LPS-induced ALI, shown by a lower lung wet/dry ratio and cytokine levels.^12^

The indication of antibiotic use are to treat bacterial infection, and the duration of treatment may range from 2 days to as long as 4–6 weeks, depending on the condition. For example, brain abscess, infective endocarditis, and pyogenic arthritis or osteomyelitis may require extended treatment durations.^13–18^ It is worthy to model the potential effects of prolonged antibiotic exposure during early life on the developing gut microbiome, growth and its subsequent impact on lung injury.

Short-term antibiotic exposure has been proved to alter gut microbial community composition.^13^ Long-term antibiotic use has also been shown to alter the composition of the microbiome in the gut and lung in experimental mice (exposure from 14 to 42 days old).^9^ The effects of prolonged antibiotic use on the gut-lung axis microbiome and subsequent ALI in rat pups remain scarcely explored.

We hypothesized that the long-term alterations to the gut microbiome induced by the prolonged antibiotic exposure since early life would affect growth and gut microbiome in immature life forms, and lead to a more severe manifestation of the subsequent lung injury at later life via the gut-lung axis. By using a rat pup model and orally administering antibiotics, the study aimed to recapitulate the potential effects of prolonged antibiotic exposure on the gut-lung axis.

## Methods

### Animal preparations

**Fig. 1:**
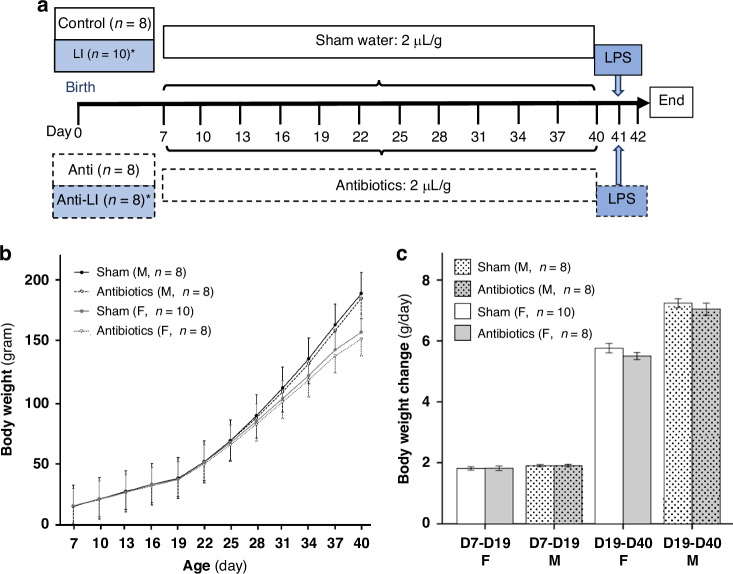
Experimental flow and the effect of antibiotics on growth patterns in experimental rat pups. **a** Experimental flow; (**b**) body weight trajectories; (**c**) daily weight changes (g/day) for both sexes across the sham and antibiotics groups. Data are depicted as mean ± SEM. *P < 0.05. Sham given sham water orally; Antibiotics given antibiotics orally; Anti antibiotics; D days old, F female rats, LI lung injury, LPS lipopolysaccharide, M male rats, SEM standard error of the mean.

Postpartum female rats and infant rats were housed in a carefully regulated environment with a temperature maintained between 20 and 22 °C, a relative humidity of 50% to 70%, and a 12-hour light-dark cycle. The designed environment ensured positive pressure with noise levels under 65 dB, creating suitable conditions for the rats. Food and water were accessible ad libitum. The delivery day was assigned as day 0 (D0), and the newborn pups remained with their mothers for breastfeeding until the 8th day post-birth (7 days old, D7). Since a 12-day-old rat pup is equivalent to a human neonate at birth, a 7-day-old rat pup is approximately equivalent to a preterm human neonate.^19^ From this point, body weights were recorded every 3 days. The pups were moved to separate cages on D21, away from their mothers, disallowing any further breastfeeding (Fig. 1a). The Institutional Animal Care and Use Committee (IACUC) of National Yang Ming Chiao Tung University provided ethical approval for all the experimental procedures involving animals (IACUC approval number: 1090234rn).

### Experimental design

Thirty-four 7-day-old Sprague-Dawley rat pups were randomly divided into four groups: Control (*n* = 8), Anti (*n* = 8), LI (*n* = 10), and Anti-LI (*n* = 8). The Anti and Anti-LI groups were orally administered antibiotics Augmentin (2 μL/g, containing Amoxicillin 400 mg and 57 mg clavulanic acid in 5 mL syrup, GlaxoSmithKline plc, Brentford, Middlesex, United Kingdom) every 3 days from day 7 (D7) to D40 (Fig. 1a). This dosing regimen was designed to achieve an amoxicillin dose of 160 mg/kg.^20,21^ The antibiotics were administered orally using a sterile pipette every 3 days to minimize disturbance of the rat pups and ensure consistent antibiotic intake on the days of weight measurements. The D7 to D40 exposure period was selected to represent prolonged antibiotic administration.^9^ The Control and LI groups received sham water during this period. On D41, the LI and Anti-LI groups underwent LPS-induced acute lung injury (ALI), and all groups were sacrificed 24 h later on D42.

### LPS-induced ALI rat model

In line with previous literature, an intra-tracheal injection procedure was replicated to simulate the LPS-induced ALI rat model.^22^ On D41, the rats of AI and Anti-LI groups underwent anesthesia with 4% isoflurane followed by intra-tracheal infusion of 5 mg/kg LPS (Escherichia coli O111:B4, γ-irradiated, BioXtra, Sigma-Aldrich, St. Louis, Missouri) diluted in sterile normal saline (0.2 mL/100 g weight).^12,22^ An otoscope was used for a better visualization of the vocal cord and trachea for LPS instillation through its cone.^23,24^ (Supplementary Fig. 1) After LPS instillation, the rats were returned to their cages upon gaining consciousness.

### Measurement of cytokines in serum and broncho-alveolar lavage fluid (BALF)

Euthanasia was performed under deep anesthesia using 5% isoflurane inhalation. Following deep anesthetization, the procedure involved exsanguination from the rat’s cardiac apex until cardiac arrest ensued. This approach ensured minimal distress and maximal humane care for the animal during the euthanasia process. The blood samples were centrifuged at 1500 rpm for 15 min at 4 °C, and the serum was stored at −80 °C.

Broncho-alveolar lavage fluid (BALF) was obtained through a mid-cervical tracheostomy using an 18-gauge cannula, with lungs lavaged using sterile saline (10 cc/kg). The total lavage procedures were conducted twice. The BALF was centrifuged at 12000 rpm for 15 min at 4 °C and immediately stored at −80 °C.

Interleukin-1 β (IL-1β) and IL-6 concentrations in BALF supernatant and serum IL-6 were measured using ELISA (R&D systems, Minneapolis, State of Minnesota, United States of America). A microplate spectrophotometer (Tecan, Männedorf, Switzerland) measured absorbance at 450 nm. Background absorbance from blank wells was subtracted before assaying sample concentrations, and duplicate measurements were taken, with average values recorded.

### Detection of C-reactive protein (CRP) in serum and BALF

CRP DuoSet ELISA gauged the CRP levels in the serum and BALF supernatant (R&D systems, Minneapolis, State of Minnesota). Absorbance was measured at a wavelength of 450 nm using a microplate spectrophotometer, after which the blank well absorbance were removed before calculating the sample concentrations. Checks of each sample were run twice, and their averages were recorded.

### Measurement of the activity of lactate dehydrogenase (LDH) in serum and BALF

The LDH levels in serum and BALF supernatant were examined with an LDH assay kit (Abcam, Cambridge, UK) as per the manufacturer’s guidelines. Tests of each sample were run twice, and their averages were notated.

### Protein measurements in serum and BALF

Bicinchoninic Acid (BCA) Protein Assays were used to evaluate total protein concentration in the serum and BALF supernatant (G-Biosciences, Page Ave, Saint Louis). Blank well absorbance was subtracted from the readings of the microplate spectrophotometer at a 562 nm wavelength, and tests of each sample were run twice, with their averages recorded.

### Histological preparation and examination of rat lung tissue

Post-harvesting, right and left lungs were fixed using a 10% neutral buffered formalin and were sectioned following embedding in paraffin into 4 µm slices stained with hematoxylin and eosin. Using a light microscope, lung tissue changes were evaluated on a scale based on seven distinct categories: alveolar inflammation, alveolar hemorrhage, interstitial hemorrhage, interstitial inflammation, edema, atelectasis, and necrosis, scored as follows: 0 - no injury; 1 - injury in < 1/4 of the field; 2 - injury spanning 1/4 to 1/2 of the field; 3 - injury in 1/2 to 3/4 of the field; 4–injury >3/4 of the field.^25^ Two laboratory personnel blindly to grouping and independently graded each lung injury category, and their mean scores were subjected to statistical analysis.

### Bacterial Microbiota Assessment of rat feces

To evaluate the bacterial microbiota, fecal matter was gathered from rat colons and stored at −80 °C for the 16S rRNA sequencing. The QIAamp PowerFecal DNA kit was used to extract bacterial DNA from the feces and BALF pellets. The V3-V4 region of the 16S rRNA gene was the focus of PCR amplification with specific primers (341 F and 806 R). Post-processing involved purification of PCR products using AMPure XP beads after the 25-cycle PCR protocol using KAPA HiFi HotStart ReadyMix (Roche).

Sequencing libraries for the 16S rRNA V3-V4 region PCR amplicons were prepared following the Illumina 16S Metagenomic Sequencing Library Preparation process. The quality of the library was checked using the Qubit 4.0 Fluorimeter and Qsep100TM system. Indexed PCR products in equal amounts were pooled for sequencing on the Illumina MiSeq platform, generating 300‐bp, paired-end reads.

### Statistical analysis

For the analysis of the animals’ data, SPSS (version 24.0) was utilized. Two-group comparisons were performed using either a *t* test or Mann–Whitney U test, whereas multiple-group comparisons employed either one-way ANOVA or Kruskal-Wallis test.

Microbiota data analysis involved several steps. Amplicon sequencing was performed, and the resulting reads were assembled with FLASH^26^ Low-quality reads (Q < 20) were discarded using QIIME.^27,28^ Chimeras were checked using UCHIME^29,30^, and the USEARCH pipeline’s UPARSE^31^ function filtered sequences at 97% identity.^32^ Taxonomy annotation was carried out using the RDP classifier algorithm^33–35^ and singletons or sequences present in only one sample were filtered out. Multiple sequence alignment and phylogenetic tree construction were conducted using PyNAST^36^ and FastTree^37,38^ in the NCBI.

For statistical analysis of the microbiota data, the following approaches were employed: (1) Data were rarefied using QIIME^39^, and alpha diversity indices such as observed operational taxonomic units (OTUs), Chao1, Abundance-based Coverage Estimator (ACE), and Shannon indexes were calculated.^40^ (2) β diversity analysis involved the computation of weighted and unweighted UniFrac distances^41,42^, supplemented by supervised PLS-DA for variance visualization. (3) Differential abundance analysis was performed using a zeroinflated Gaussian log-normal model.^43^ Statistical significance was assessed using welch’s *t* test^44^, LEfSe analysis,^45^ and STAMP software.

A *P*-value of less than 0.05 was considered statistically significant in all analyses.

## Results

Our experiment encompassed thirty-four 7-day-old Sprague-Dawley rat pups, 18 females and 16 males, weighing between 13–18 grams (15.7 ± 1.1 g) (Table 1).

**Table 1:** Comparison of the α diversity index of gut microbiota among four study groups.

Group	Control *n* = 8	Anti *n* = 8	LI *n* = 10	Anti-LI *n* = 8
Sex				
Female, n (%)	5 (62.5)	3 (37.5)	5 (50)	5 (62.5)
Male, n (%)	3 (37.5)	5 (62.5)	5 (50)	3 (37.5)
Body weight				
D7W, g	15.4 (0.3)	15.9 (0.4)	15.4 (0.4)	14.8 (0.4)
D40W, g	167 (5.7)	176 (7.2)	177 (6.4)	163 (6.0)
α diversity				
ACE^a^	464 (15.9)	365 (11.8)*	430 (36.0)	312 (26.2)*§
Chao1^b^	467 (16.5)	370 (13.6)*	432 (37.7)	316 (27.7)*§
Richness^c^	423 (15.8)	325 (11.1)*	390 (36)	270 (21.5)*§
Shannon^d^	6.17 (0.2)	5.62 (0.1)*	5.61 (0.4)	4.64 (0.3) *†§

Data were expressed as number (%) or mean (SEM).

*p < 0.05 compared with the Control group, †*p* < 0.05 compared with the Anti group, §*p* < 0.05 compared with the LI group.

*SEM* standard error of the mean, *D7W* body weight of 7-day-old rats, *D40W* body weight of 40-day-old rats, *n* number, *g* gram, *%* percentage, *Anti* the rats with antibiotics exposure, *LI* the rats with lung injury only, *Anti-LI* the rats with antibiotics exposure and lung injury.

^a^ACE (Abundance-based coverage estimator index) estimates the total number of unique species or operational taxonomic units (OTUs), by using the abundance distribution of rare and abundant taxa to estimate total richness.

^b^Chao1 estimates the total number of unique species or OTUs, including rare/unobserved taxa, by using the number of singletons and doubletons to extrapolate the true richness.

^c^Richness index counts the total number of unique species/OTUs observed.

^d^Shannon index measures both the richness and the evenness of the community by incorporating the relative abundance of each species/OTU.

### Weight analysis

The weight growth trajectories were similar across all subgroups, including both female and male rats in the Sham and Antibiotic-exposed groups, from D7 to D19 (Fig. 1b). However, beginning on D19, the weights of the sham and antibiotic-exposed groups started to diverge, with the sham groups demonstrating gradually increasing weight gains compared to the antibiotic-exposed groups in both sexes. However, there was no significant difference at any day point between sham and antibiotics-exposed groups for both sexes (*P* > 0.05) (Fig.1b, c).

### Gut microbiome

The Anti and Anti-LI groups showed significant reductions in fecal ACE, Choa1, species richness, and Shannon index compared to the Control and LI groups respectively. Notably, the Shannon index of Anti-LI was lower than the Anti group (Table 1 and Supplementary Fig. 2) (*p* < 0.05).

**Fig. 2:**
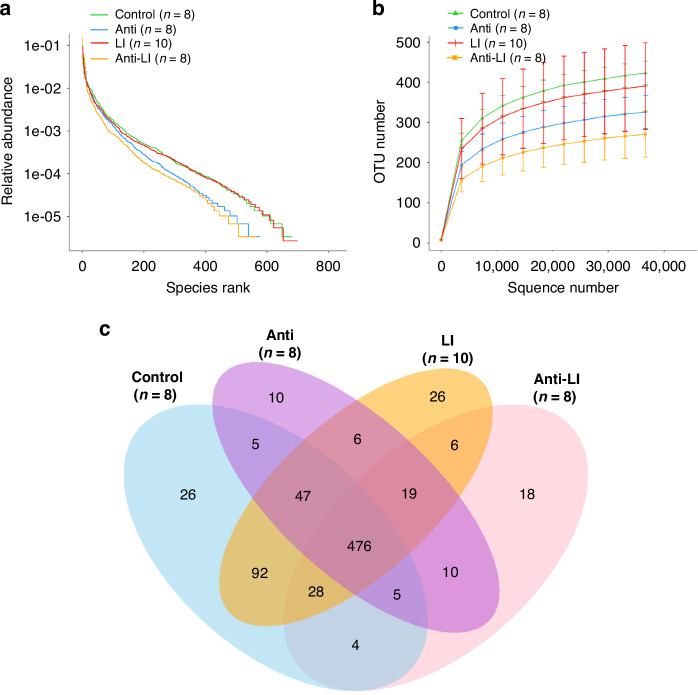
Comparative analysis of fecal microbial community and diversity in experimental rat pups across four groups: control, antibiotics (anti), lung injury (LI), and combined antibiotics and lung injury (anti-LI). **a** Rank Abundance Curve demonstrates species richness and evenness. The Anti and Anti-LI groups displayed lesser species richness and evenness compared to the Control and LI groups. **b** Rarefaction curve, indicating OTU number among individual fecal samples, plots the Control group with the highest number of OTUs, followed by the LI, Anti, and Anti-LI groups. **c** Venn diagram illustrates OTU distribution across the groups. With 778 OTUs in total across all groups, 476 OTUs (61%) were shared. OTU Operational Taxonomic Unit.

Figure 2a depicts the Rank Abundance Curve, which provides a visual representation of the richness and evenness of species within the groupings.^46,47^ As shown, the species richness and evenness of the Anti and Anti-LI groups were lower than the Control and LI groups (Fig. 2a). Figure 2b presents the Rarefaction Curve generated by randomly subsampling a certain number of sequencing reads from each sample and counting the operational taxonomic units (OTUs).^40^ The OTU numbers were highest in the Control group, followed by the LI, Anti, and Anti-LI groups, respectively (Fig. 2b). Figure 2c shows the Venn diagrams based on the OTU cluster analysis results.^48^ Each circle represents a group, with overlapping areas indicating the number of common OTUs. Of the 778 total OTUs found, 61% were shared by the four groups (Fig. 2c). The Control group contained 683 OTUs (69.6% shared), the Anti group contained 578 OTUs (82.4% shared), the LI group contained 700 OTUs (68% shared), and the Anti-LI group had 566 OTUs (84.1% shared). It can be observed that the Anti and Anti-LI groups had fewer OTUs compared to the Control and LI groups. Additionally, Fig. 2c also illustrates the OTU sharing proportions of the four study groups. As shown, both the Anti and Anti-LI groups demonstrated a higher sharing proportion with other groups compared to each of the Control and LI groups. This suggests that antibiotic exposure reduced the number of unique OTUs within the affected groups but increased the proportion of OTUs shared with other groups.

**Fig. 3:**
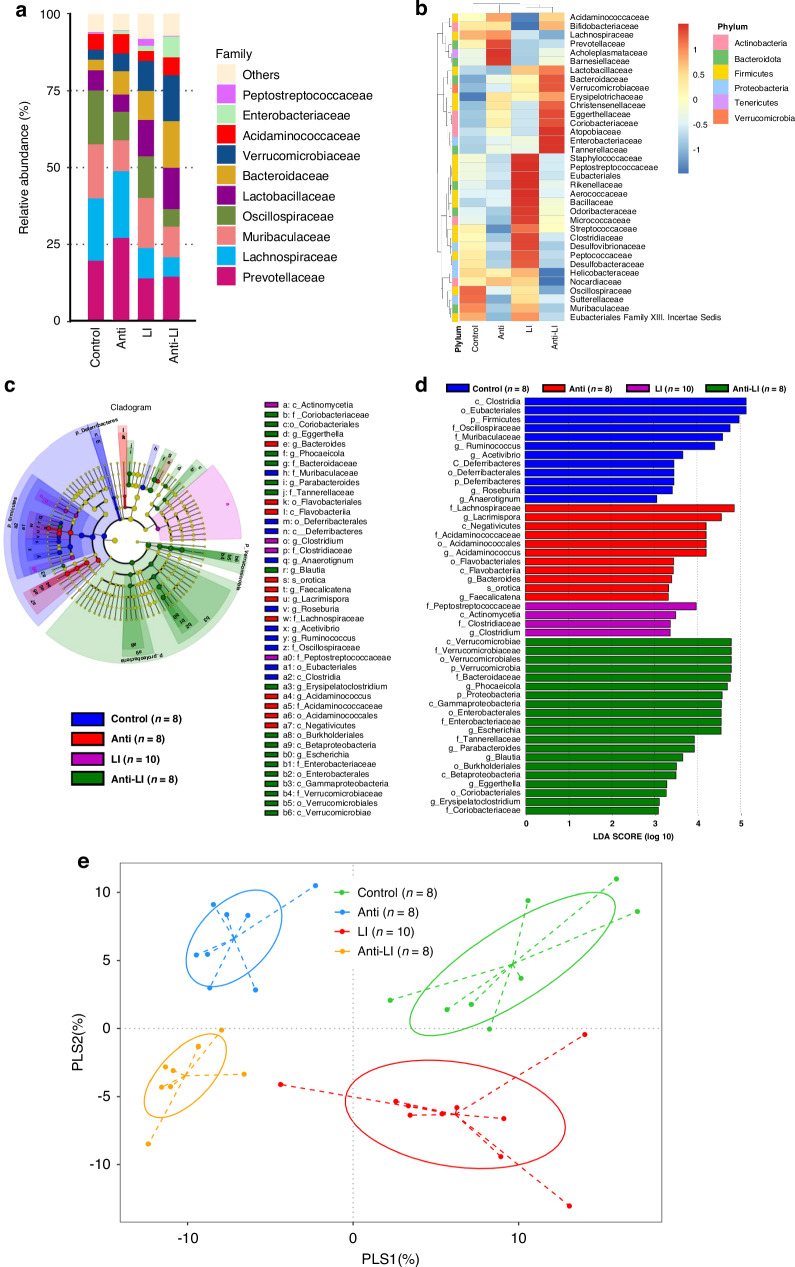
Analysis of fecal bacterial diversity and relative abundance in experimental rat pups belonging to four study groups: control, antibiotics (anti), lung injury (LI), and combined antibiotics and lung injury (anti-LI). **a** Represents the top 10 bacterial families in abundance and rest aggregated as “Others”. Significant shifts in notable bacterial families are observed across the groups. **b** Heatmap illustrating the relative abundance of the top thirty-five bacterial families, revealing distinct microbial profiles for each study group. LEfSe analysis identifies 47 bacterial characteristic taxa as significantly varying across groups at different taxonomic levels, depicted in a Cladogram (**c**) and corresponding LDA scores chart (**d**). **e** Partial Least Squares Discriminant Analysis (PLS-DA) of β-diversity across groups, indicating higher β-diversity in Control and LI groups compared to their counterparts Anti and Anti-LI. Β-diversity in the Anti-LI group was marginally lower than the Anti group, with no significant differences observed between Control and LI groups. LEfSe denotes Linear Discriminant Analysis Effect Size, LDA stands for Linear Discriminant Analysis, PLS-DA represents Partial Least Squares Discriminant Analysis, c class, o order, f family, g genus, s species, log logarithm.

Significant shifts in bacterial abundance appeared when comparing antibiotics-exposed groups to their counterparts. Specifically, *Muribaculaceae* and *Oscillospiraceae* decreased while *Bacteroidaceae* and *Verrucomicrobiaceae* increased in each antibiotics-exposed group (Fig. 3a).

LEfSe analysis revealed distinct microbiota abundances across the groups. For instance, *Deferribacteres* and *Clostridia* were more abundant in the Control group, while *Betaproteobacteria*, *Gammaproteobacteria*, and *Verrucomicrobiae* were more abundant in the Anti-LI group (Fig. 3c, d).

Beta diversity was greater in Control and LI groups when compared to their antibiotics-exposed counterparts (Fig. 3e).

**Fig. 4:**
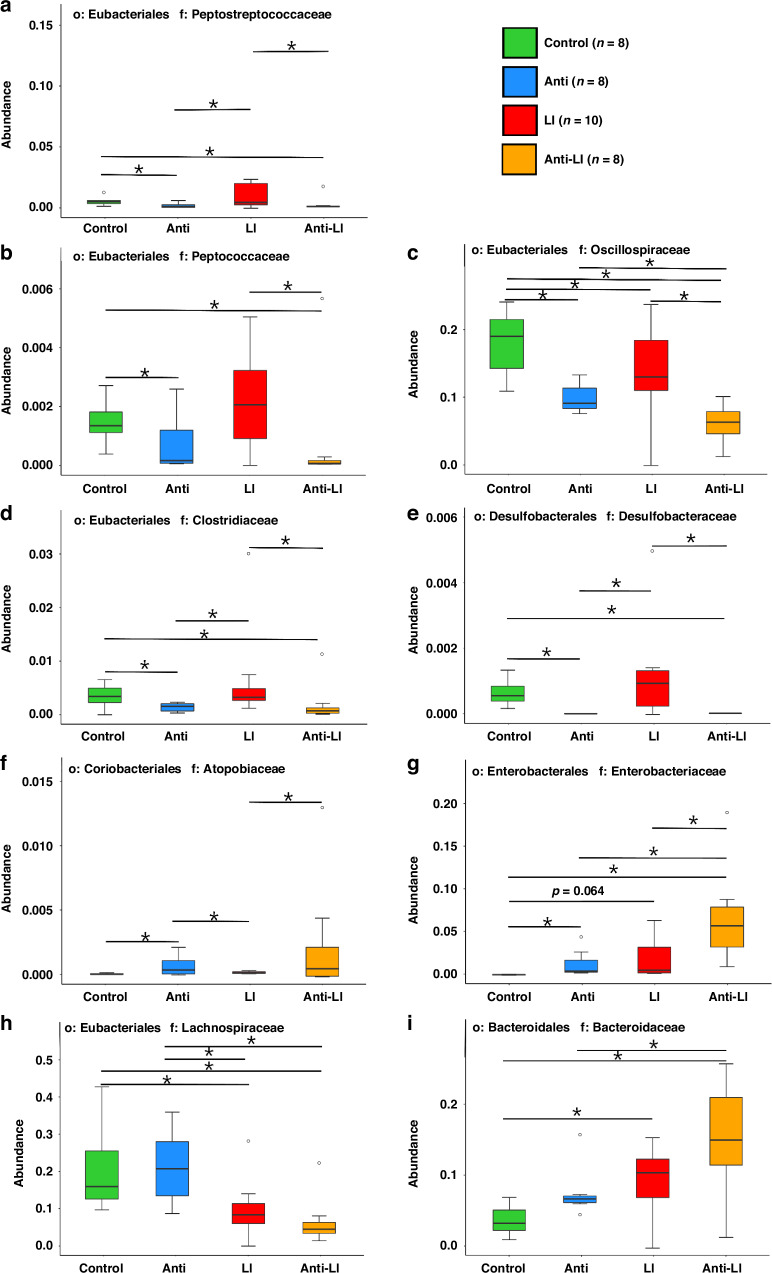
Comparative analysis of significant variations in bacterial abundance at the family level in fecal samples of rat pups across four study groups: control, antibiotics (anti), lung injury (LI), and combined antibiotics and lung injury (anti-LI), as shown by MetagenomeSeq analysis. **a** *Peptostreptococcaceae*. **b** *Peptococcaceae*. **c** *Oscillospiraceae*. **d** *Clostridiaceae*. **e** *Desulfobacteraceae*. **f** *Atopobiaceae*. **g** *Enterobacteriaceae*. **h** *Lachnospiraceae*. **i** *Bacteroidaceae*. Each panel represents a specific microbial family and indicates significant changes in their abundance among 4 study groups. * *P* < 0.05, o order, f family.

Statistical analysis revealed decreased abundance of *Peptostreptococcaceae*, *Peptococcaceae*, *Oscillospiraceae*, *Clostridiaceae*, and *Desulfobacteraceae* in Antibiotic groups. At the same time, abundance of *Atopobiaceae* and *Enterobacteriaceae* was noticeably higher in these groups (Fig. 4a–g). Changes post-LPS intra-tracheal instillation also showed significant changes in bacterial abundance (Fig. 4c, g–i).

### LPS-induced ALI

**Fig. 5:**
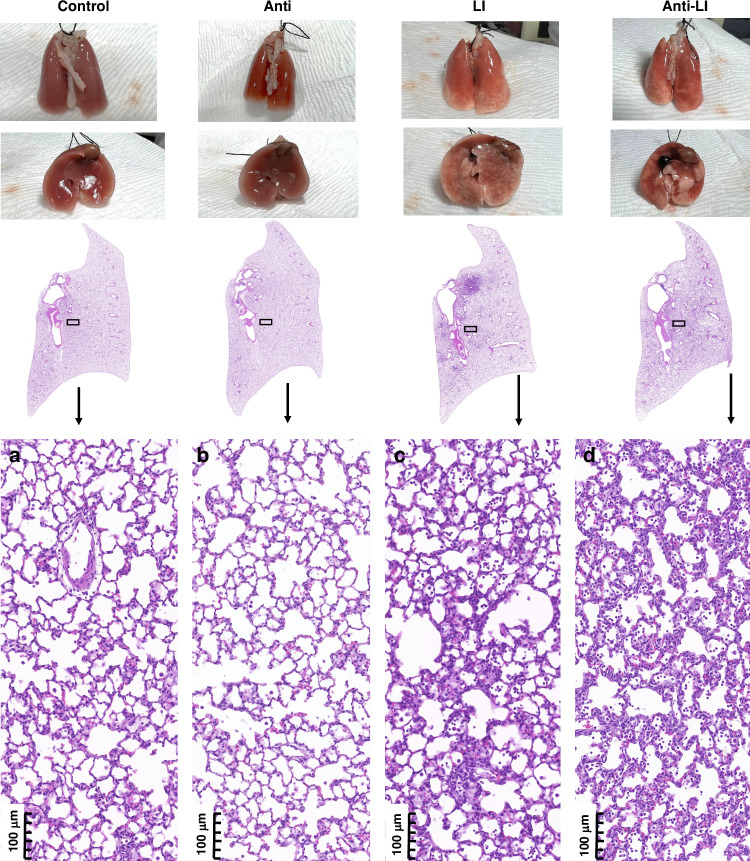
Hematoxylin and eosin-stained lung tissue sections from experimental rat pups in four study groups: Control, Antibiotics (Anti), Lung Injury (LI), and combined Antibiotics and Lung Injury (Anti-LI), magnified 200 times. **a** Control group. **b** Anti group. **c** LI group. **d** Anti-LI group. µm micrometer.

Figure 5 illustrates the gross and histological images of the experimental groups. The lungs of the Control and Anti groups displayed even and smooth characteristics, whereas the LI and Anti-LI groups presented marked uneven lung surfaces, with clear signs of consolidation, inflammation, and hemorrhage, notably at dependent locations.

Histologically, there was a significant increase in inflammatory cell infiltration and structural aberrations in the LI and Anti-LI groups when compared to their counterparts (Fig. 5a–d). The assessment of lung injury scores revealed both LI and Anti-LI groups had drastically higher scores for alveolar and interstitial inflammation, hemorrhage, atelectasis, necrosis, and overall lung injuries compared to the Control and Anti groups (*P* < 0.05). Yet, no significant discrepancies were found in the scores for individual categories or total lung injury scores when comparing the Control to Anti or LI to Anti-LI groups (Table 2) (*P* > 0.05).

**Table 2:** Lung injury scores of experimental animals among four study groups.

Group	Control *n* = 8	Anti *n* = 8	LI *n* = 10	Anti-LI *n* = 8
Lung injury score (sub-items)				
Alveolar				
Alveolar inflammation	1.2 (0.5)	1 (0.3)	4 (7)*§	5.8 (5.5)*§
Alveolar hemorrhage	0.7 (1.3)	1 (0.7)	1.8 (3.8)*	1 (0.5)
Interstitial				
Interstitial hemorrhage	1.2 (0.8)	1.5 (0.5)	3.5 (2.1)*§	3.5 (0.5)*§
Interstitial inflammation	2 (0.5)	1.5 (1)	3.2 (1.7)*§	3.5 (0.8)*§
Edema	0 (0)	0 (0.3)	0 (0.12)	0 (0)
Atelectasis	1 (0.3)	1 (0)	1.5 (0.6)*	1.3 (1)*
Necrosis	1.5 (0.8)	1 (0.7)	2.2 (0.7)*§	1.5 (0.5)§
Lung injury score (sum)	7.2 (1.8)	7 (3.6)	16.2 (13.7)*§	17.8 (5.6)*§

Data are presented as median (IQR).

*p < 0.05 compared with the Control group.

§*p* < 0.05 compared with the Anti group.

*IQR* interquartile range, *n* number, *Anti* rats exposed to antibiotics, *LI* rats exclusively with lung injury, *Anti-LI* rats exposed to antibiotics and with lung injury.

**Fig. 6:**
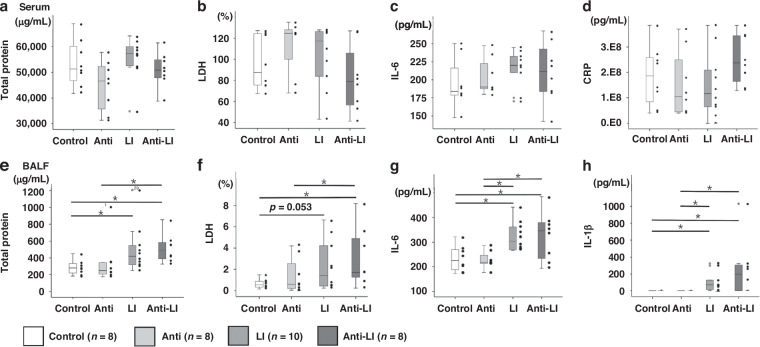
Scatterbox plots representing the levels of key inflammation-related substances in both serum and bronchoalveolar lavage fluid (BALF) supernatants from experimental rat pups across four groups: control, antibiotics (Anti), lung injury (LI), and combined antibiotics and lung injury (Anti-LI). **a** Total protein in serum. **b** LDH proportion in serum. **c** IL-6 content in serum. **d** CRP concentration in serum. **e** Total protein content in BALF supernatant. **f** LDH proportion in BALF supernatant. **g** IL-6 content in BALF supernatant. **h** IL-1β concentration in the supernatant of BALF. Serum levels of all 4 inflammatory substances showed no significant intergroup differences (**a**–**d**), but BALF analyses revealed significantly elevated total protein, LDH, IL-6, and IL-1β in the LI group when compared to the Control group, and in the Anti-LI group when compared to the Anti group (**e**–**h**). *P < 0.05, LDH lactate dehydrogenase, IL-6 interleukin 6, IL-1β interleukin-1 β, CRP C-reactive protein.

In terms of inflammation-related substances in BALF and serum, no significant differences were observed across all groups for serum substances (*P* > 0.05). However, in BALF the total protein, LDH, IL-6, and IL-1β levels were significantly higher in the LI and Anti-LI groups than in their respective Control and Anti groups (*P* < 0.05). Yet, there were no significant differences between the LI and Anti-LI groups in BALF substances (Fig. 6a–h) (*P* > 0.05).

## Discussion

In this rat pup experiment, antibiotic-exposed groups exhibited significantly reduced fecal microbiota diversity and notable alterations in bacterial abundance. Non-antibiotic groups experienced slightly higher, though non-significant, weight gain compared to antibiotic-exposed groups. The severity of subsequent acute lung injury (ALI) did not significantly differ between the groups.

### Antibiotic impact on gut microbiome

Consistent with previous studies, our research shows that Amoxicillin-Clavulanic acid exposure reduces both α and β diversity in the gut microbiome.^3,49,50^ We also demonstrated decreased species richness and evenness, and fewer unique but more shared OTUs among antibiotic-exposed groups. These results highlight the significant impact of Amoxicillin-Clavulanic acid on gut microbial diversity, revealing the ecological effects of antibiotic use.

In terms of the microbial community abundance within the intestine, Amoxicillin-Clavulanic acid increased the richness of *Atopobiaceae* and *Enterobacteriaceae* families. This supports prior findings that suggest Amoxicillin-Clavulanic acid augments the abundance of *Enterobacteriaceae*.^51^

Focusing on gut microbiota richness reduction, we found Amoxicillin-Clavulanic acid significantly lowered the abundance of families like *Peptostreptococcaceae*, *Peptococcaceae*, *Oscillospiraceae*, *Clostridiaceae*, and *Desulfobacteraceae*. These results align with previous studies pointing out that Amoxicillin reduces *Clostridiaceae*^51^ and *Firmicutes*^3,49^ richness. Notably, *Peptostreptococcaceae*, *Peptococcaceae*, *Oscillospiraceae*, and *Clostridiaceae* belong to the *Firmicutes* phylum, known to facilitate energy absorption and weight gain.^52^ The decline in *Firmicutes* observed in our experiments may explain the trend, although not statistically significant, of lower weights in antibiotic-exposed rats. Other studies also report Amoxicillin exposure reduces body weight.^53^ Possible mechanisms for this weight reduction include decreased microbial diversity leading to impaired nutrient absorption, altered gut hormonal signals affecting appetite and metabolism, and inflammation-related changes disrupting normal metabolic processes.

### Weight changes during study periods

Although we observed a trend of reduced weight gain in the antibiotic-exposed animals after D19, but the weight growth trajectories did not show statistically significant differences between antibiotic-exposed and unexposed rats. This lack of significance may be attributed to the study’s endpoint at 6 weeks of age. A study by Tulsturb MV et al. observed weight differences from 9 to 14 weeks when antibiotic exposure ended at 4 weeks, indicating that group disparities may intensify over time.^54^ Extending observations beyond 6 weeks in our study might have revealed significant weight differences.

We identified a turning point on the nineteenth day, evident in the body weight growth charts of female and male rats starting on the seventh day after birth. Daily weight gain after the nineteenth day exceeded that before, likely due to the dietary shift at 21 days, when rats are weaned from mother’s milk to dry feed. This dietary revision likely impacted nutritional intake, leading to deviations in caloric gain.

### LPS-induced ALI and gut microbiome

This study showed that LPS-induced lung inflammation altered gut microbiota, increasing *Enterobacteriaceae* and *Bacteroidaceae* while reducing *Oscillospiraceae* and *Lachnospiraceae*. This aligns with previous studies showing a rise in *Enterobacteriaceae* during sepsis^55^ and a loss of *Lachnospiraceae* due to impaired gastrointestinal motility and intestinal damage.^56^

Sepsis significantly diminishes gut microbial diversity.^55^ Previous research has shown that intra-tracheal LPS administration impairs intestinal α diversity, β diversity, uniformity, and OTU numbers via the gut-lung axis.^57^ Our study, using a lower LPS dose and a 24-hour post-LPS period before sacrifice, saw a reduction in OTU numbers but no significant changes in gut α (Table 1) or β diversity. The mild methodology likely limited the observation of pronounced intestinal diversity changes, unlike studies with higher LPS doses and longer ALI duration.^57^

### Antibiotic impact on LPS-induced ALI

Our study revealed that prolonged early-life exposure to amoxicillin-clavulanic acid did not affect BALF inflammation markers or lung injury scores after ALI in later life of rats. However, it significantly altered gut microbiota diversity and richness. Despite consistent systemic inflammation markers (total protein, LDH, IL-6, IL-1β) across groups, antibiotic exposure reduced β diversity, species richness, evenness, and significantly lowered the Shannon index post LPS-induced ALI. The inflammation-related serum biomarkers remained unchanged, likely due to the relatively mild lung injury severity and short post-instillation duration.

### Study limitations

Our study enhances our understanding of prolonged early-life antibiotic exposure, but several limitations exist. Controlled lab conditions limit real-world generalizability where diverse factors affect neonatal physiology and gut microbiota. Using a single antibiotic, Amoxicillin-Clavulanic acid, in a specific dosing may not represent typical clinical scenarios. Relatively mild ALI induced may not fully capture prolonged antibiotic impact on pulmonary injury. The absence of microbiome analysis in BALF limits our evaluation of the gut-lung axis. Future research should include diverse settings, antibiotic varieties, severe ALI models, dual-site microbiome analysis, and longer weight monitoring.

## Conclusion

Prolonged early-life antibiotic exposure in this rat pup model significantly reduced gut microbiome diversity and exhibited a trend towards lower weight gain, though this was not statistically significant. Importantly, antibiotic exposure did not significantly exacerbate subsequent LPS-induced acute lung injury. Future studies with longer observation periods, more severe lung injury models, and a broader assessment of the gut-lung axis are warranted to better understand these relationships.

## Supplementary information

Supplementary Figures

## Data Availability

All datasets generated or analyzed during the presented study are available from the corresponding author upon reasonable request.
