# Supplementary material for: Prolonged early-life antibiotic exposure alters gut microbiota but does not exacerbate lung injury in a rat pup model
Source: Pediatr Res. 2025 Apr 9;98(2):723–33. doi: 10.1038/s41390-025-03924-2 (PMC12454151; doi:10.1038/s41390-025-03924-2)
Supplement: Supplementary file 1 — Supplementary Figures [file 41390_2025_3924_MOESM1_ESM.pdf]

SUPPLEMENTARY FIGURES

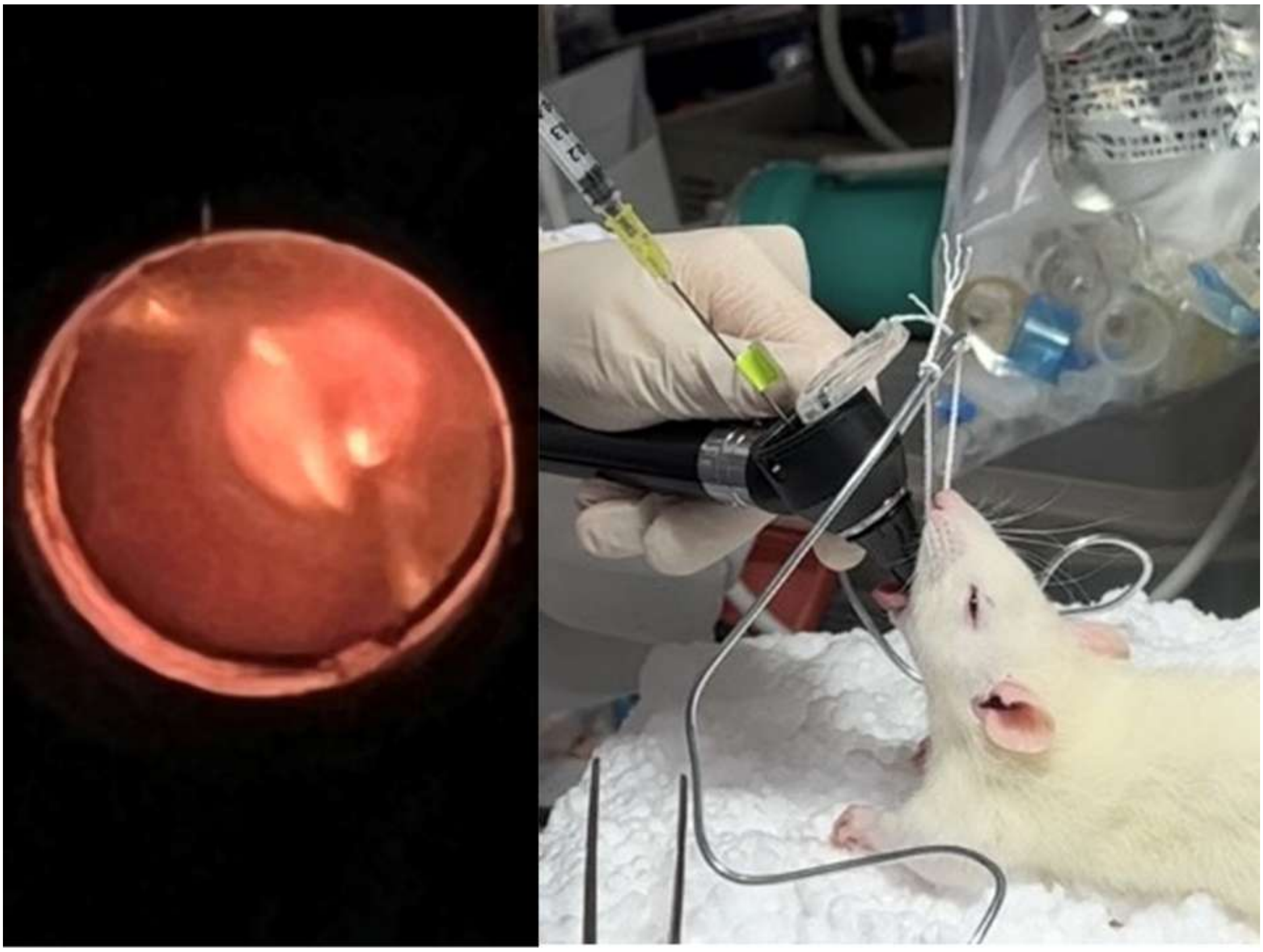

Fig. 1. Visualization and administration of Lipopolysaccharide (LPS) into the trachea of Rat Pups. Left: A detailed view of the tracheal opening observed through an otoscope, highlighting the clear anatomical structures of the trachea. Right: A procedural image showing the use of an otoscope to administer Lipopolysaccharide (LPS) directly into the trachea of a rat pup. A syringe is used into the otoscope cone, ensuring accurate delivery of LPS into the tracheal lumen. The rat pup is positioned securely, and the procedure is conducted under aseptic conditions to ensure both accuracy and animal welfare.

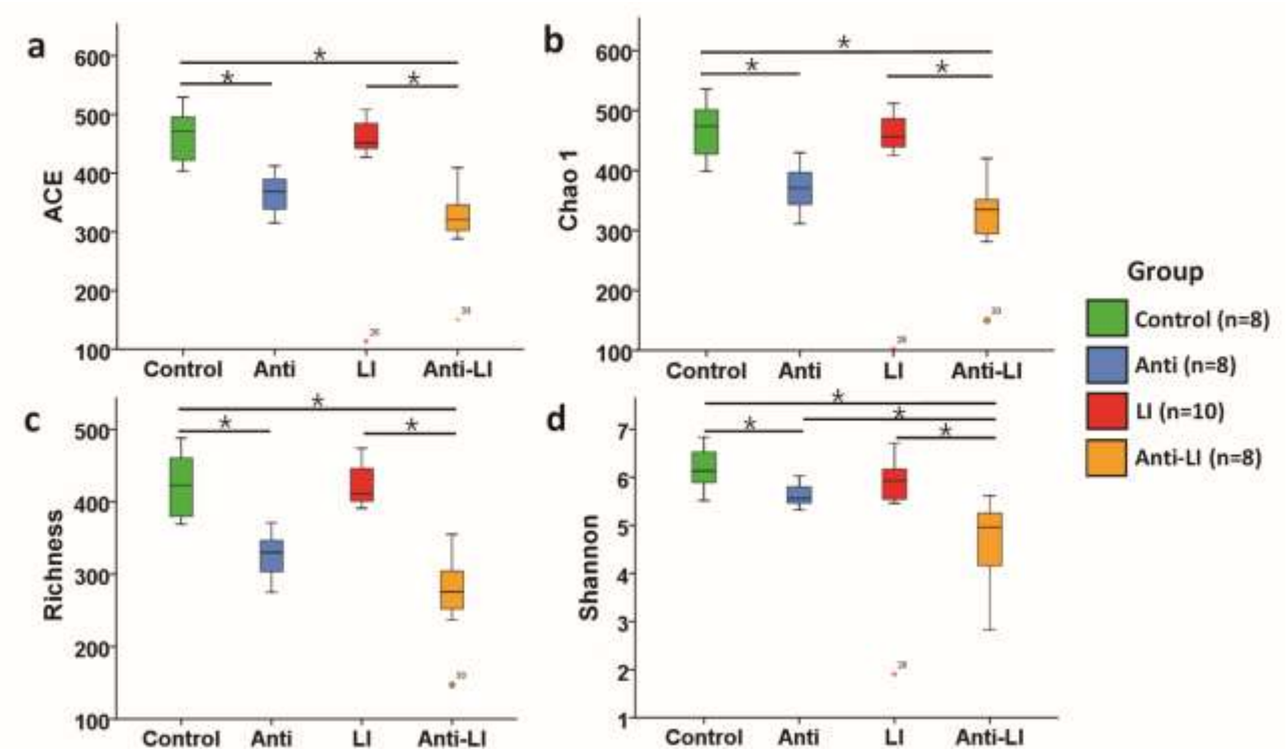

Fig. 2. Comparative analysis of microbial diversity across four study groups: Control, Antibiotics (Anti), Lung Injury (LI), and combined Antibiotics and Lung Injury (Anti-LI). Box plots represent (a) ACE (Abundance-based coverage estimator index), (b) Chao1, (c) Richness, and (d) Shannon indices. The Control group (green, n=8) serves as the baseline, while the Anti (blue, n=8), LI (red, n=10), and Anti-LI (orange, n=8) groups represent different treatment conditions. Statistically significant differences among groups are indicated by asterisks ($P < 0.05$). The data demonstrate significant reductions in microbial diversity in the Anti and Anti-LI groups compared to the Control and LI groups, as indicated by all four diversity indices (ACE, Chao1, Richness, and Shannon).
